# Adaptation and Validation of the Traditional Chinese Family Caregiver Medication Administration Hassle Scale

**DOI:** 10.1093/geroni/igaf122.382

**Published:** 2025-12-31

**Authors:** Te-Lien Ku, Shu-Ying Wu, Wei-Chu Chie, Yen-Ming Huang

**Affiliations:** University of Wisconsin-Madison, Madison, Wisconsin, United States; Sijhih Cathay General Hospital, New Taipei City, New Taipei, Taiwan (Republic of China); National Taiwan University, Taipei, Taipei, Taiwan (Republic of China); National Taiwan University, Taipei City, Taipei, Taiwan (Republic of China)

## Abstract

The Family Caregiver Medication Administration Hassle Scale (FCMAHS) measures the stress family caregivers experience in managing medication for older adults. However, its applicability to Chinese-speaking caregivers in Taiwan remains uncertain. This study adapted the FCMAHS into Traditional Chinese (FCMAHS-TC). We conducted a two-phase cross-sectional design: Phase I involved bidirectional translation and expert evaluation, while Phase II used a convergent mixed-methods field test. A purposive sample of 138 family caregivers in Taiwan completed the FCMAHS-TC, and 12 participants were interviewed. Exploratory factor analysis (EFA) determined the factor structure of the scale. Validity and reliability were assessed through content validity, internal consistency, and test-retest reliability. Caregiver interviews were audio-recorded, transcribed verbatim, and analyzed deductively using content analysis to triangulate evidence for scale adaptability by exploring caregivers’ medication management experiences and associated hassles. Expert evaluation confirmed excellent content validity, with an item-and scale-level content validity index of 1.0. The EFA revealed a four-factor structure explaining 59.57% of the variance, including Information Seeking and Sharing (11 items), Scheduling Logistics (7 items), Medication Filling and Prescription Management (3 items), and Safety Issues (3 items). Reliability analysis showed strong internal consistency (Cronbach’s alpha = 0.94) and good test-retest reliability (intra correlation coefficient = 0.78). Qualitative findings supported the items and factor structure while highlighting Care Coordination as a distinct hassle for Taiwanese family caregivers. The FCMAHS-TC demonstrates strong psychometric properties and cultural adaptability for the Taiwanese population, helping researchers and healthcare providers better understand family caregivers’ medication management challenges and design targeted interventions.

